# Latent profile analysis and determinants of social isolation among older adults in China

**DOI:** 10.3389/fpubh.2026.1845790

**Published:** 2026-08-21

**Authors:** Xiaoxing Lai, Zhiyuan Zhang, Yonghua Cai, Yang Li, Rui Sun, Shuai Zhang, Kexin Zhang, Hongdi Du, Shuxian Li, Jingwei Hao, Xiaopeng Huo

**Affiliations:** 1 Department of Neurology, Peking Union Medical College Hospital, Chinese Academy of Medical Sciences and Peking Union Medical College, Beijing, China; 2 Department of Nursing, Peking Union Medical College Hospital, Chinese Academy of Medical Sciences and Peking Union Medical College, Beijing, China; 3 Department of Healthcare, Peking Union Medical College Hospital, Chinese Academy of Medical Sciences and Peking Union Medical College, Beijing, China

**Keywords:** determinants, frailty, latent profile analysis, older adults, social isolation

## Abstract

**Objective:**

This study aimed to identify social isolation profiles among older adults in China using latent profile analysis and to explore their associations with demographic and health factors.

**Methods:**

A cross-sectional study was conducted between July and December 2023 in the Chaoyang and Dongcheng districts of Beijing, China. Participants aged 60 years or older were recruited through convenience sampling from six communities. Latent profile analysis was used to classify social isolation patterns, and multivariate logistic regression models to assess associated factors.

**Results:**

529 participants were included. Three social isolation profiles were identified: the mild social isolation subgroup (*n* = 239, 45.2%), the moderate social isolation subgroup (*n* = 213, 40.3%), and the severe social isolation subgroup (*n* = 77, 14.5%). Comparing mild to severe social isolation, multivariate analysis revealed that older adults living alone without caregiver had significantly higher odds of severe social isolation compared to those living with children (OR = 0.099, 95% CI: 0.034–0.377, *P* < 0.001). Frailty (OR = 0.141, 95% CI: 0.039–0.588, *P* = 0.033), severe ADL dependence (OR = 0.249, 95% CI: 0.145–0.454, *P* = 0.048), and moderate to severe IADL dependence (OR = 0.448 to 0.307, *P* = 0.046, *P* = 0.017) were also significantly associated with severe social isolation. Similarly, in the comparison between the moderate and severe subgroups, compared to those living with children, older adults living alone without caregiver were more likely to experience severe social isolation (OR = 0.199, 95% CI: 0.078–0.328, *P* = 0.001). In terms of functional status, severe ADL dependence (OR = 0.152, 95% CI: 0.061–0.505, *P* = 0.015) and moderate to severe IADL dependence (OR = 0.231 to 0.128, *P* = 0.045, *P* = 0.033) were significantly associated with severe social isolation.

**Conclusion:**

Older adults living alone without caregiver, with frailty, and with functional dependence are at significantly higher risk of severe social isolation. Family network integrity emerged as the primary dimension differentiating severe social isolation from mild-to-moderate isolation, which may be linked to a quantitative gradient of overall network restriction. Understanding the specific factors contributing to different profiles could inform tailored support strategies to enhance social engagement and quality of life in this population.

## 1 Introduction

Social isolation among older adults has emerged as a critical public health concern due to its substantial impact on both mental and physical wellbeing (1). Social isolation is defined as an objective, measurable lack of meaningful and sustained communication or contact with social connections, characterized by a restricted social network with a low number of social interactions and weak network density (2, 3). In China, the prevalence of social isolation among middle-aged and older individuals is estimated at 16.48%, with particularly high rates observed among women and those living in rural areas (4). Comparable rates have been reported in European countries, underscoring the widespread nature of this issue (5).

Extensive evidence indicates that social networks built through sustained interpersonal interactions provide crucial emotional, instrumental, and informational support, thereby mitigating many of the adverse effects associated with aging (6, 7). The importance of social connectedness cannot be overstated, as it is closely linked to improved psychological resilience, enhanced cognitive functioning, and better physical health outcomes (8).

However, the prevalence of social isolation among older adults in China remains a cause for concern. Several contributing factors have been identified, including age-related declines in physical and cognitive function, as well as shifting familial and societal roles resulting from changes in traditional family structures (9, 10). Moreover, the rapid advancement of digital technology, while offering new avenues for communication, has also inadvertently widened the digital divide. Many older adults face difficulties in adopting new technologies due to barriers such as limited digital literacy and access, which may exacerbate their sense of disconnection (11, 12).

The cumulative impact of these challenges has been associated with markedly increased health risks, including a 29% higher risk of incident coronary heart disease, a 50% higher risk of developing dementia, and a 32% higher risk of all-cause mortality among socially isolated older adults (13–15). Despite growing awareness, most existing research remains focused on population-level estimates, often overlooking the heterogeneity of social isolation experiences among older individuals (16–18). Furthermore, traditional assessment tools typically rely on aggregated scale scores, which may obscure nuanced patterns and individual differences in social isolation (16, 19). To address these gaps, this study applies Latent Profile Analysis (LPA), a person-centered statistical technique that identifies distinct subgroups based on multidimensional response patterns related to social isolation. This approach enables a more refined understanding of the typologies of social isolation in older adults.

The aim of this study is to identify latent profiles of social isolation among older adults using LPA and to examine the sociodemographic, health-related, and psychosocial factors associated with each profile.

## 2 Methods

### 2.1 Study design and participants

This cross-sectional study used convenience sampling to recruit older adults from six communities in Chaoyang and Dongcheng districts of Beijing, China between July and December 2023.

Inclusion criteria included: age ≥60 years; participants who provided informed consent, were voluntary, and were capable of normal communication and cooperation to complete the study.

Exclusion criteria included: psychiatric disorders; severe organic diseases involving the heart, brain, liver, or kidneys; or being in the terminal stage of illness.

The approval for the study protocol was obtained from the Ethics Committee of Peking Union Medical College Hospital (Approval No. ZS-2943), and all participants signed informed consent forms.

### 2.2 Data collection

General Information Questionnaire: this questionnaire was developed to collect baseline sociodemographic characteristics and clinical history variables. The questionnaire items were adapted from established national aging survey protocols widely used in Chinese geriatric research, including the Chinese Longitudinal Healthy Longevity Survey (CLHLS) and the China Health and Retirement Longitudinal Study (CHARLS) (20, 21).

This questionnaire was used to collect demographic data including age, sex, occupation, education, marital status, living arrangement, number of children, household economic status, and method of medical expense payment. It also included clinical history variables such as multimorbidity, polypharmacy, family history of chronic illness, and smoking and alcohol consumption.

Lubben Social Network Scale (LSNS-12) (22, 23): this 12-item scale was used to assess social isolation, consisting of two subscales: family network (items 1–6) and friend network (items 7–12). It evaluates the number of family members or friends with whom the individual can maintain contact, converse, or seek help. Response options include “none,” “1,” “2,” “3–4,” “5–8,” and “9 or more,” scored from 0 to 5. Total scores range from 0 to 60, with lower scores indicating more limited social networks and a higher likelihood of social isolation. A dichotomous classification was applied using a cutoff score of < 19 to identify participants meeting criteria for social isolation with the Chinese version of the LSNS (24, 25). The Chinese version of the LSNS exhibited good internal consistency, with a Cronbach's α of 0.792 (24, 25).

Fried Frailty Phenotype (FFP) (26): based on the frailty cycle model, this scale assesses physical frailty using five criteria: unintentional weight loss, slowed walking speed, reduced grip strength, decreased physical activity, and fatigue. Each criterion is assigned 1 point if present. A total score of 3 or more indicates frailty; 1–2 points indicates pre-frailty; and 0 points indicates non-frailty.

Montreal Cognitive Assessment (MoCA) (27): developed by Nasreddine et al. (27), the MoCA is used to assess cognitive function. It was translated into Chinese by Wang et al. (28) for use among populations with varying degrees of cognitive impairment. The Cronbach's α coefficient of the scale is 0.836 (28). The MoCA includes 12 items across eight domains. Each item is scored 1 point for a correct answer and 0 points for an incorrect answer, yielding a total score ranging from 0 to 30. A score below 26 indicates cognitive impairment, with higher scores reflecting better cognitive performance.

Barthel Index (BI) (29): the BI is used to evaluate Basic Activities of Daily Living (BADL), including feeding, transfers between bed and wheelchair, personal hygiene, toileting, bathing, ambulation (45 m), stair climbing, dressing, bowel control, and bladder control. Total scores range from 0 to 100. Scores of 100 indicate independence; 75–95, mild dependence; 50–70, moderate dependence; 25–45, severe dependence; and 0–20, total dependence.

Lawton-Brody Instrumental Activities of Daily Living (IADL) Scale (30): this scale assesses eight areas: shopping, transportation, cooking, housekeeping, laundry, telephone use, medication management, and financial handling. The maximum score is 8 points, with higher scores indicating greater functional independence. A score of 8 reflects full independence; scores below 8 indicate varying degrees of dependence. Specifically, scores of 6–7 represent mild dependence; 3–5, moderate dependence; and ≤ 2, severe dependence. The Cronbach's α coefficient of the scale is 0.880.

Prior to data collection, all research staff underwent standardized training. Researchers established communication with community health personnel to explain the study objectives and procedures and to obtain cooperation. Before administering the questionnaire, the researchers provided standardized instructions explaining the study's purpose, significance, and survey procedures. All data were collected directly. Upon completion of each questionnaire, responses were immediately reviewed. For unclear responses, the researchers followed up to clarify and verify the information. Completed questionnaires were collected on site. Any questionnaire with more than 20% missing data was excluded from analysis. Double data entry and cross-checking were performed to ensure accuracy. A total of 540 questionnaires were distributed. Five individuals withdrew after signing informed consent, and six questionnaires were incomplete. Thus, 529 valid questionnaires were collected, yielding a response rate of 98.0%. Double data entry and verification were performed to ensure data accuracy.

### 2.3 Sample size calculation

According to the sample size estimation method for regression analysis (31), which recommends a sample size 10 to 20 times the number of independent variables, and considering a 20% attrition rate, a minimum of 225 participants was required for 18 predictors.

### 2.4 Statistical analysis

For normally distributed continuous variables, we described data as mean ± standard deviation; for non-normally distributed variables, data were reported as median and interquartile range. Categorical and ordinal variables were described using frequencies and percentages. Latent profile analysis was conducted using Mplus 8.3 to classify individuals based on distinct scoring patterns. Akaike Information Criterion (AIC), Bayesian Information Criterion (BIC), and sample-size adjusted BIC (aBIC) are indices that help us identify the goodness-of-fit of the model. These values do not have fixed cutoff points; instead, we compared the values across models with different numbers of subgroups and selected the model with the low value as the best-fitting one. Model fit was assessed using AIC, BIC, and aBIC, with smaller values indicating better fit. The Bootstrap Likelihood Ratio Test (BLRT) and the Lo-Mendell-Rubin (LMR) test helped us determine whether adding one more profile (subgroup) to the model meaningfully improves how well the model fits the data. For example, these tests compared a model with 3 classes (k) against a model with 2 classes (k−1). A significant result (*P* < 0.05) means that the model with 3 classes (k) fits the data significantly better, and we should keep the model with 3 classes (k). A non-significant result (*P* > 0.05) means the model with 2 classes (k−1) is sufficient. Entropy values, ranging from 0 to 1, were used to evaluate classification precision, with higher values indicating better accuracy. Descriptive statistics, chi-square tests, analysis of variance, and Kruskal–Wallis *H*-tests were performed using SPSS version 26.0. A multivariate logistic regression analysis was performed to ascertain factors associated with the different latent profiles of social isolation. *P* < 0.05 was considered statistically significant.

## 3 Results

### 3.1 Baseline characteristics

A total of 529 older adults aged 64 to 100 years (mean age 81.35 ± 6.25) were included in the study. The majority of participants were female (321, 60.7%), previously employed as workers (269, 50.9%), and married (353, 66.7%), while 169 (31.9%) were widowed. Regarding living arrangements, 253 (47.8%) resided with a spouse, 156 (29.5%) lived with children, 74 (14.0%) lived alone without caregiver, and 46 (8.7%) lived alone with caregiver. Most participants were covered by urban employee health insurance (315, 59.5%), and 142 (26.8%) received government-funded care. Monthly household income varied significantly, with 318 (60.1%) earning between 6,500 and 25,000 yuan, and 144 (27.2%) earning less than 2,500 yuan. Multimorbidity was present in 364 (68.8%) of 529 participants; 221 (41.8%) reported a family history of chronic disease, and 285 (53.9%) had polypharmacy (Table 1).

**Table 1:** Univariate analysis of latent profiles of social isolation among older adults.

Variables	Total	Mild social isolation	Moderate social isolation	Severe social isolation	*P*
		(*n* = 239)	(*n* = 213)	(*n* = 77)	
Age	529	81.06 ± 6.24	81.91 ± 6.48	80.71 ± 5.55	0.223
Sex					0.034
Female	321	139 (43.3%)	125 (38.9%)	57 (17.8%)	
Male	208	100 (48.1%)	88 (42.3%)	20 (9.6%)	
History of occupation					0.060
Cadre	178	82 (46.0%)	74 (41.6%)	22 (12.4%)	
Worker	269	126 (46.8%)	104 (38.7%)	39 (14.5%)	
Farmer	69	29 (42.0%)	26 (37.7%)	14 (20.3%)	
Cultural worker	7	0 (0.0%)	7 (100.0%)	0 (0.0%)	
Other	6	2 (33.3%)	2 (33.3%)	2 (33.4%)	
Highest educational attainment					0.171
No formal education	43	13 (30.2%)	20 (46.5%)	10 (23.3%)	
Primary school	88	44 (50.0%)	30 (34.1%)	14 (15.9%)	
Junior high school	128	66 (51.5%)	44 (34.4%)	18 (14.1%)	
Senior high school	88	34 (38.6%)	40 (45.5%)	14 (15.9%)	
College degree or above	182	82 (45.1%)	79 (43.4%)	21 (11.5%)	
Marital status					0.000
Never married	2	1 (50.0%)	0 (0.0%)	1 (50.0%)	
Married	353	173 (49.0%)	161 (45.6%)	19 (5.4%)	
Divorced	5	2 (40.0%)	0 (0.0%)	3 (60.0%)	
Widowed	169	63 (37.3%)	52 (30.8%)	54 (31.9%)	
Living arrangement					0.000
Living alone without caregiver	74	0 (0.0%)	3 (4.1%)	71 (95.9%)	
Living alone with caregiver	46	20 (43.5%)	26 (56.5%)	0 (0.0%)	
Living with spouse	253	131 (51.8%)	118 (46.6%)	4 (1.6%)	
Living with children	156	88 (56.4%)	66 (42.3%)	2 (1.3%)	
Number of children	529	3.00 (2.00, 3.00)	2.00 (1.00, 3.00)	2.00 (1.00, 3.50)	0.023
Medical payment method					0.148
Government-funded care	142	65 (45.8%)	62 (43.7%)	15 (10.5%)	
Urban health insurance	315	144 (45.7%)	124 (39.4%)	47 (14.9%)	
Rural health insurance	71	30 (42.3%)	27 (38.0%)	14 (19.7%)	
Out-of-pocket payment	1	0 (0.0%)	0 (0.0%)	1 (100.0%)	
Monthly household income					0.393
< 2,500 yuan	144	61 (42.4%)	66 (45.8%)	17 (11.8%)	
2,500–6,500 yuan	67	27 (40.3%)	28 (41.8%)	12 (17.9%)	
6,500–25,000 yuan	318	151 (47.5%)	119 (37.4%)	48 (15.1%)	
Multimorbidity					0.830
No	165	75 (45.4%)	64 (38.8%)	26 (15.8%)	
Yes	364	164 (45.1%)	149 (40.9%)	51 (14.0%)	
Family history of chronic disease					0.520
No	307	139 (45.3%)	119 (38.8%)	49 (15.9%)	
Yes	221	100 (45.2%)	93 (42.1%)	28 (12.7%)	
Smoking and alcohol use history					0.977
No	504	228 (45.2%)	203 (40.3%)	73 (14.5%)	
Yes	25	11 (44.0%)	10 (40.0%)	4 (16.0%)	
Polypharmacy					0.520
No	244	116 (47.5%)	92 (37.7%)	36 (14.8%)	
Yes	285	123 (43.2%)	121 (42.4%)	41 (14.4%)	
Fried frailty phenotype (FP)					0.003
Non-frail	220	110 (50.0%)	85 (38.6%)	25 (11.4%)	
Pre-frailty	216	102 (47.2%)	86 (39.8%)	28 (13.0%)	
Frailty	93	27 (29.0%)	42 (45.2%)	24 (25.8%)	
Montreal cognitive assessment (MoCA)					0.766
Normal cognition	381	170 (44.6%)	157 (41.2%)	54 (14.2%)	
Cognitive impairment	148	69 (46.6%)	56 (37.8%)	23 (15.6%)	
ADL					0.000
Independent	249	126 (50.6%)	103 (41.4%)	20 (8.0%)	
Mild dependence	203	89 (43.8%)	85 (41.9%)	29 (14.3%)	
Moderate dependence	59	21 (35.6%)	21 (35.6%)	17 (28.8%)	
Severe dependence	18	3 (16.7%)	4 (22.2%)	11 (61.1%)	
IADL					0.000
Independent	297	157 (52.9%)	105 (35.3%)	35 (11.8%)	
Mild dependence	92	35 (38.0%)	47 (51.1%)	10 (10.9%)	
Moderate dependence	122	43 (35.2%)	56 (45.9%)	23 (18.9%)	
Severe dependence	18	4 (22.2%)	5 (27.8%)	9 (50.0%)	

Significant differences in social isolation profile membership were identified across subgroups defined by sex, marital status, living arrangement, number of children, frailty status, and both basic and instrumental activities of daily living (*P* < 0.05). Detailed results are presented in Table 1.

Among 74 older adults living alone without caregiver, 71 (96.0%) exhibited severe social isolation. This subgroup comprised 18 females and 56 males, with 55 (74.3%) being widowed, 15 (20.3%) married, three (4.0%) divorced, and one (1.4%) unmarried. Regarding physical status, 27 (36.5%) showed no signs of frailty, 27 (36.5%) were pre-frail, and 20 (27.0%) were frail. In terms of cognitive function, 56 (75.7%) had normal cognition, while 18 (24.3%) had cognitive impairment. Regarding socioeconomic status of this subgroup (*n* = 74), 18 participants (24.3%) reported a monthly household income of less than 2,500 yuan, 10 participants (13.5%) had a monthly household income between 2,500 and 6,500 yuan, and 46 participants (62.2%) had a monthly household income ranging from 6,500 to 25,000 yuan. In terms of educational attainment, 11 participants (14.9%) had no formal schooling, 12 (16.2%) had completed primary education, 19 (25.7%) had completed junior secondary education, 11 (14.8%) had completed senior secondary education, and 21 (28.4%) had attained a university degree or higher. Regarding pre-retirement occupations, the subgroup comprised 21 (28.4%) cadres, 39 (52.7%) workers, and 14 (18.9%) farmers.

### 3.2 Scores for social isolation, frailty, cognitive function, BADL, and IADL

The Lubben Social Network Scale scores ranged from 5 to 40, with a mean of 28.17 (±8.67). Using the LSNS-12 cutoff of < 19 points, a total of 239 participants (45.2%) met the criteria for social isolation. Fried frailty scores varied from 0 to 5, with a mean of 1.26 (±1.29), and 309 participants (58.4%) were classified as frail. The MoCA scores ranged from 9 to 30, yielding a mean score of 25.35 (±4.43), with 148 participants (30.0%) exhibiting cognitive impairment. Barthel Index scores ranged from 5 to 100, with a mean of 90.02 (±14.79); 166 participants (31.4%) demonstrated limitations in BADL. Finally, IADL scores ranged from 0 to 8, with a mean of 6.65 (±1.91), and 232 participants (43.9%) displayed dependence in IADL.

### 3.3 Latent profile analysis of social isolation

Beginning with class 1 model, latent profile models encompassing one to four classes were estimated (Table 2). As the number of classes increased, the values of the AIC, BIC, and aBIC decreased, while Entropy values fluctuated. Although the class 2 model exhibited relatively high entropy (0.885), its AIC (3,688.936), BIC (3,706.020), and aBIC (3,693.323) were elevated. The class 4 model demonstrated a lower AIC (3,640.953) than class 3 (3,643.077); however, the BLRT *P*-value was not statistically significant (*P* > 0.05). This marginal non-significance suggests that the class 4 model does not represent a statistically robust improvement over the class 3 model. Furthermore, the class 4 model yielded two very small classes with class probabilities of 0.076 and 0.078, respectively, which compromised both statistical stability and substantive interpretability. The class 3 model demonstrated the lowest BIC (3,668.703) and aBIC (3,649.657), and both LMRT *P*-value and BLRT *P*-value were statistically significant (*P* < 0.001), supporting its statistical validity and practical utility. Taking into account the profile analysis fit indices (e.g., AIC, BIC, aBIC), the BLRT significance test, and class probability distributions, the class 3 model was ultimately selected as the optimal model.

**Table 2:** Model fit indices for latent class models.

Class	AIC	BIC	aBIC	Entropy	P (LMRT)	P (BLRT)	Class probability
1	3,788.775	3,797.317	3,790.968	—	—	—	1
2	3,688.936	3,706.020	3,693.323	0.885	< 0.001	< 0.001	0.153/0.867
3	3,643.077	3,668.703	3,649.657	0.804	< 0.001	< 0.001	0.403/0.452/0.146
4	3,640.953	3,675.121	3,649.727	0.821	0.010	0.051	0.076/0.452/0.078/0.395

K, number of freely estimated parameters; AIC, akaike information criterion; BIC, Bayesian information criterion; aBIC, adjusted Bayesian information criterion; Entropy, information entropy; LMR, Lo-Mendell-Rubin likelihood ratio test; BLRT, bootstrap likelihood ratio test.

**Figure 1:**
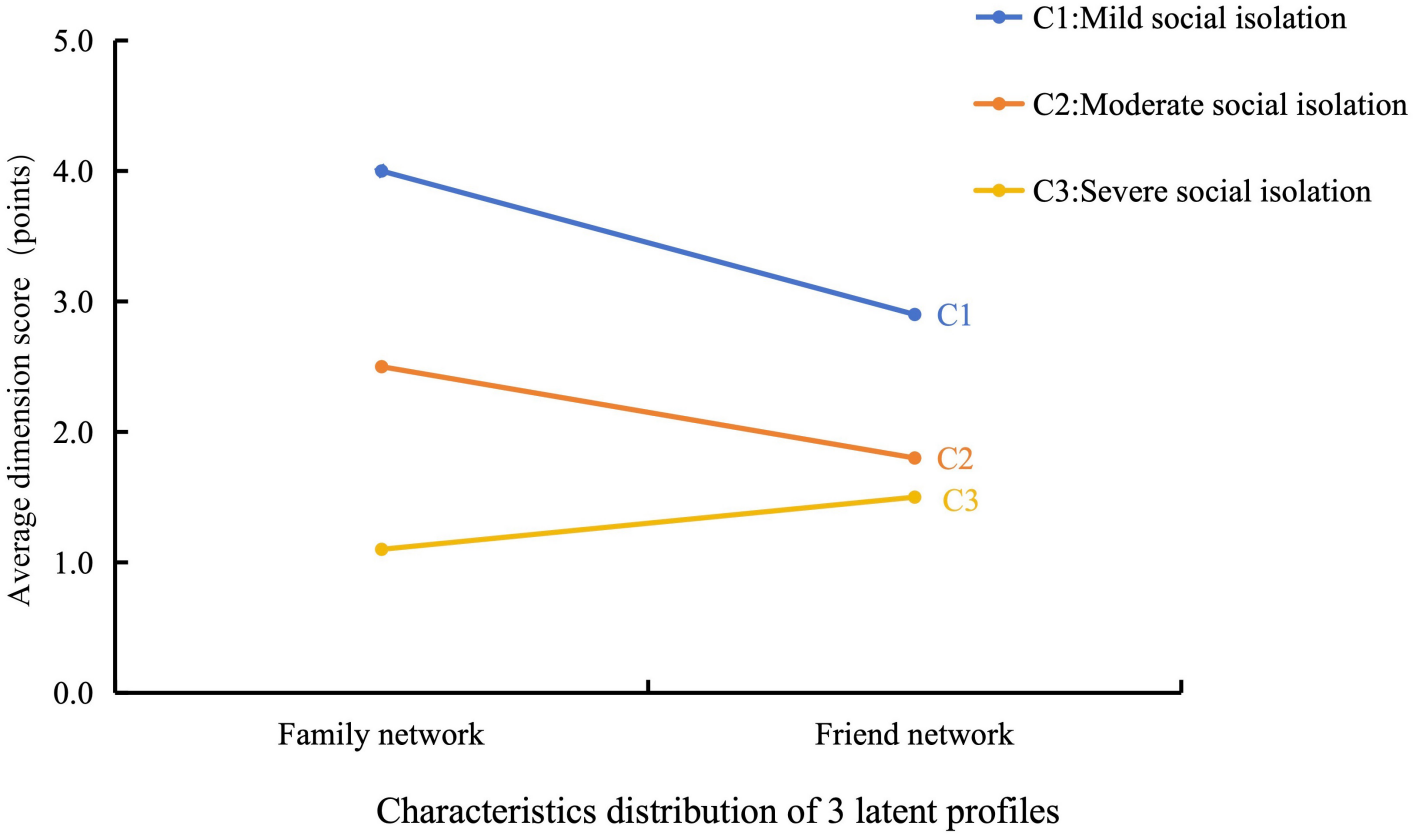
Three latent profiles of social isolation among older adults. Data are mean item scores for each subscale of the Lubben Social Network Scale (LSNS-12), rated 0 (none) to 5 (≥9 contacts). Family network: items 1–6; friend network: items 7–12. C1, mild social isolation (*n* = 239); C2, low friendship network–moderate social isolation (*n* = 213); C3, low family network–severe social isolation (*n* = 77).

Based on the results of latent profile analysis, the study population was categorized into three distinct classes (Figure 1 and Table 2). C1 included 239 individuals (45.2%) who exhibited relatively high scores on the LSNS and was therefore designated as Mild Social Isolation Subgroup. C2 comprised 213 individuals (40.3%) whose LSNS scores were moderate overall, with comparatively low scores in the friend network dimension; this subgroup was termed Moderate Social Isolation Subgroup. C3 consisted of 77 individuals (14.5%) who had the lowest LSNS scores overall, including the lowest scores in both family and friend network domains, and was thus identified as Severe Social Isolation Subgroup. The Severe Social Isolation Subgroup had a mean total LSNS score of 11.62 (SD = 3.74, range: 5–18), the Moderate Social Isolation Subgroup had a mean of 25.94 (SD = 2.74, range: 19–30), and the Mild Social Isolation Subgroup had a mean of 35.48 (SD = 2.95, range: 31–40).

### 3.4 Logistic analysis of latent profiles of social isolation

Independent variables included those exhibiting statistically significant differences in the univariate analysis. The dependent variable was the latent profile of social isolation, categorized into mild (C1), moderate (C2), and severe (C3), with the Severe Social Isolation Subgroup serving as the reference category. In the context of comparing mild to severe social isolation, multivariate analysis revealed that older adults living alone without caregiver had significantly higher odds of severe social isolation compared to those living with children (OR = 0.099, 95% CI: 0.034–0.377, *P* < 0.001). Frailty (OR = 0.141, 95% CI: 0.039–0.588, *P* = 0.033), severe ADL dependence (OR = 0.249, 95% CI: 0.145–0.454, *P* = 0.048), and moderate to severe IADL dependence (OR = 0.448 to 0.307, *P* = 0.046, *P* = 0.017) were also significantly associated with severe social isolation.

Similarly, in the comparison between moderate and severe social isolation, compared to those living with children, older adults living alone without caregiver were significantly more likely to experience severe social isolation (OR = 0.199, 95% CI: 0.078–0.328, *P* = 0.001). In terms of functional status, severe ADL dependence (OR = 0.152, 95% CI: 0.061–0.505, *P* = 0.015), moderate IADL dependence (OR = 0.231, 95% CI: 0.166–0.583, *P* = 0.045), and severe IADL dependence (OR = 0.128, 95% CI: 0.057–0.392, *P* = 0.033) were significantly associated with severe social isolation. Other variables showed no statistically significant associations (*P* > 0.05; Table 3).

**Table 3:** Multivariate logistic regression analysis of factors associated with latent profiles of social isolation among older adults.

Variables	Reference category	OR	95%CI	*P*
Mild social isolation vs. severe social isolation				
Number of children		1.477	0.689–3.164	0.316
Sex (female)	Male	1.050	0.172–6.399	0.958
Marital status	Widowed			
Never married		36.562	0.106–53.799	0.669
Married		1.119	0.128–9.793	0.919
Divorced		35.410	0.579–64.355	0.611
Living arrangement	Living with children			
Living alone with caregiver		0.045	0.017–10.326	0.535
Living alone without caregiver		0.099	0.034–0.377	0.000
Living with spouse		0.325	0.022–4.884	0.416
Frailty status	Non-frail			
Pre-frailty		0.417	0.244–1.613	0.058
Frailty		0.141	0.039–0.588	0.033
ADL	ADL independent			
Mild dependence		0.668	0.314–2.269	0.485
Moderate dependence		0.491	0.205–1.134	0.062
Severe dependence		0.249	0.145–0.454	0.048
IADL	IADL independent			
Mild dependence		0.701	0.533–1.625	0.096
Moderate dependence		0.448	0.215–0.577	0.046
Severe dependence		0.307	0.206–0.511	0.017
Moderate social isolation vs. severe social isolation				
Number of children		1.127	0.531–2.394	0.755
Sex (female)	Male	1.107	0.184–6.655	0.911
Marital status	Widowed			
Never married		0.203	0.104–4.855	0.110
Married		1.466	0.176–12.192	0.723
Divorced		0.023	0.006–4.883	0.579
Living arrangement	Living with children			
Living alone without caregiver		0.199	0.078–0.328	0.001
Living alone with caregiver		6.398	0.774–10.339	0.669
Living with spouse		0.416	0.028–6.175	0.524
Frailty status	Non-frail			
Pre-frailty		0.239	0.104–1.974	0.109
Frailty		0.103	0.031–13.612	0.103
**ADL**	ADL independent			
Mild dependence		0.508	0.388–1.831	0.172
Moderate dependence		0.328	0.212–1.069	0.072
Severe dependence		0.152	0.061–0.505	0.015
IADL	IADL independent			
Mild dependence		0.436	0.315–1.227	0.062
Moderate dependence		0.231	0.166–0.583	0.045
Severe dependence		0.128	0.057–0.392	0.033

## 4 Discussion

This study investigated social isolation among 529 community-dwelling older adults in China, revealing a significant prevalence of social isolation, with 239 participants (45.2%) meeting the established criteria. Through LPA, three distinct social isolation profiles were identified: mild social isolation, moderate social isolation, and severe social isolation. This classification enhances the understanding of social dynamics within aged populations and underscores the necessity for tailored interventions to address varying degrees of isolation. This person-centered approach aligns with Machielse's (32) social isolation typology, which similarly identified heterogeneous subgroups of socially isolated older adults based on the quality and quantity of social relationships (32). However, Machielse's typology emphasized qualitative dimensions, whereas our latent profiles were derived from quantitative LSNS scores. Methodologically akin to the study by Lunar et al. (33), this study conducted sociodemographic stratification to examine profile distribution differences in social isolation among older adult subgroups with varying demographic characteristics, including age, gender, occupation, marital status, and household income. Based on these typological characteristics, differentiated, mechanism-oriented, and precision intervention programs could be designed. The research paradigm has evolved from understanding heterogeneity to quantifying heterogeneity, and this study lays the groundwork for further outcome prediction and intervention design.

### 4.1 Social isolation profiles and integrated intervention strategies

The Mild Social Isolation Subgroup (*n* = 239, 45.2%) was characterized by relatively strong family and friend networks. The Moderate Social Isolation Subgroup (*n* = 213, 40.3%) maintained limited family ties, engagement with friends and the community. In contrast, the Severe Social Isolation Subgroup (*n* = 77, 14.6%) exhibited low scores across both domains, reflecting comprehensive disconnection.

Living alone without caregiver was a strong predictor of severe social isolation. Among 74 participants (96.0%) who lived alone without caregiver, nearly all were classified as severely isolated. This finding aligns with previous research demonstrating that the absence of family support significantly reduces emotional and instrumental resources, thereby increasing the risk of social disconnection (34, 35).

This pattern must be understood within the specific context of Chinese society, where the family remains the cornerstone of older adults' social support system. However, the traditional extended family structure has undergone fundamental transformation, shifting toward nuclear families and replacing multi-generational co-residence with the “4–2–1” family pattern (36). The “4–2–1” family pattern refers to a family configuration that emerged in China as a direct consequence of the one-child policy (officially implemented from 1980 to 2015). A single child might potentially support two parents and four grandparents, creating severe caregiving burden and time constraints. Consequently, rapid urbanization and declining family sizes may futher exacerbate isolation among those without co-residing children or caregiver (37–39). These structural changes generate a hypothesis. When family structures are compromised, friend and community networks may serve a compensatory function; however, severe isolation represents a state where both compensatory mechanisms have failed. The severe isolation observed in 96.0 % of older adults living alone without caregiver cannot be understood solely through individual health deficits. Rather, it reflects a convergence of decline in family support and systemic service gaps. Consistent with Shin and Park's (40) findings on social network typologies, their study demonstrated that individuals with family-centered networks showed different loneliness-depression trajectories compared to those with friend-centered or diversified networks (40). Identifying these subgroups enables public health practitioners and policymakers to develop interventions that target specific deficits. For instance, community engagement strategies may be particularly beneficial for those who retain family support but lack social networks outside the household. A concrete example of community-based intervention could be found in Beijing's “Silver-Age Volunteer Service Posts.” Under the coordination of sub-district offices and community service centers, older adults are recruited as Waste Sorting Supervisors, guiding residents in proper waste classification, while accumulating credits that could be exchanged for community services or goods.

By positioning older adults as service providers rather than passive recipients, this mechanism reconstructs their sense of social value and simultaneously expands non-family social networks through structured, recurring contact with diverse community members. The community interventions, such as Silver-Age Volunteer Service Posts programs, are designed precisely to address these structural barriers, though current reach remains insufficient for this highly vulnerable subgroup.

The observed profiles highlight possible directions for future interventions, which should include strategies that not only encourage family involvement but also establish formal community support systems, such as home visit programs, social clubs, or volunteer networks, to support older adults living alone.

### 4.2 Functional status and community engagement

Dependence in basic and instrumental activities of daily living (ADL/IADL) was significantly associated with more severe social isolation profiles. Individuals with severe functional limitations were more likely to fall into the Moderate or Severe Social Isolation Subgroups. Physical impairments may restrict mobility, limit participation in social activities, and reduce opportunities to maintain friendships, even when family support is present (41, 42). Longitudinal studies have also demonstrated that declines in ADL and IADL predict subsequent increases in social isolation and loneliness (41, 42). However, given the cross-sectional design of the present study, we cannot determine the directionality of this relationship. Future longitudinal research is needed to clarify the temporal sequence and causal pathways linking functional limitations and social isolation. In this study, dependence in ADL and IADL was significantly associated with a higher likelihood of being in the Severe Isolation Subgroup. This association underscores the importance of addressing functional limitations in interventions aimed at reducing social isolation among older adults, while recognizing that the observed relationship may be bidirectional. To reduce the risk of social isolation, community-based compensatory mechanisms could be considered. For example, “virtual families” can be constructed through neighborhood pairing organized by the community to compensate for the loss of family functions; additionally, community canteens could be opened to reconstruct daily interaction scenarios akin to family settings. It should be pointed out that the severe social isolation subgroup comprised only 77 individuals (14.6%) in this study, which limits statistical power and increases the risk of incidental findings and requires replication in larger, more diverse cohorts.

### 4.3 Frailty and its bidirectional relationship with social isolation

Frailty was another significant factor associated with social isolation. Frailty, a state of increased vulnerability to stressors, has a complex and potentially bidirectional relationship with social isolation. Non-frail participants were more likely to belong to the Mild Social Isolation Subgroup, whereas frail individuals were overrepresented in the Moderate and Severe Social Isolation Subgroups. Frail individuals may be less able to participate in social activities due to reduced physiological reserves, fatigue, and decreased mobility, while social isolation itself may accelerate the development or progression of frailty (43). Frailty limits physiological reserves and mobility, potentially reducing participation in social activities, while social isolation itself may accelerate frailty through decreased physical activity and psychosocial stimulation (44–46). However, due to the cross-sectional design of this study, it remains unclear whether frailty precedes social isolation, social isolation precedes frailty, or whether both conditions mutually reinforce each other over time. These findings suggest the potential importance of integrating frailty screening into routine geriatric care. Early identification and multicomponent interventions—including physical exercise, nutritional support, and psychosocial engagement—may mitigate both frailty and social isolation, ultimately improving quality of life (43, 47).

### 4.4 Limitations

This study has several limitations. First, the participants were limited to community-dwelling older adults, and no data were collected from rural or hospitalized populations. And whether the participants had regular access to or utilization of community support services were not explored. Second, the survey was confined to urban districts in Beijing, which may limit the generalizability of the findings. Third, the study was cross-sectional in design, precluding any longitudinal assessment of changes in social isolation over time. Fourth, the LSNS measures only the quantity and frequency of contact with family members and close friends, and does not assess social networks derived from community participation (23). Group membership and civic engagement constitute a distinct and meaningful dimension of social integration. This measurement gap may have introduced a conservative bias in our findings. The friend domain of the LSNS captures only close friendships and may miss broader community-based non-familial connections (e.g., neighbors, volunteer contacts, club members). Future studies incorporating community participation measures—such as the social participation items used by Machielse (32)—are needed to disentangle close friendship from broader community integration. Another limitation of this study is the exclusion of individuals with psychiatric conditions, which may restrict the generalizability of our findings to the full spectrum of aging adults. Future research should expand the sample size, incorporate multi-center designs, and collaborate with community health authorities to conduct comprehensive assessments of social isolation among older adults.

## 5 Conclusions

This study employed latent profile analysis to adopt a person-centered approach, classifying older adults' social isolation according to distinct characteristic patterns, thereby facilitating the implementation of targeted interventions to improve their social isolation status. This study identified distinct patterns of social isolation among community-dwelling older adults in China, including mild social isolation, moderate social isolation, and severe social isolation. Older adults living alone without caregivers were more likely to experience severe social isolation. Those with moderate or severe dependence in ADL and IADL were more likely to fall into the Moderate Isolation Subgroup. In contrast, the absence of frailty was associated with a higher probability of belonging to the Mild Social Isolation Subgroup. Family network integrity emerged as the primary dimension differentiating severe social isolation from mild-to-moderate isolation, which may be linked to a quantitative gradient of overall network restriction. These cross-sectional findings point to potential implications for individualized interventions based on specific social isolation profiles. Continuous monitoring, tailored support, and personalized management strategies should be implemented to mitigate social isolation in older adults. Longitudinal follow-up is also needed to better understand temporal trends and transitions in social isolation status.

## Data Availability

The raw data supporting the conclusions of this article will be made available by the authors, without undue reservation.
